# Direct TLR2 Signaling Is Critical for NK Cell Activation and Function in Response to Vaccinia Viral Infection

**DOI:** 10.1371/journal.ppat.1000811

**Published:** 2010-03-12

**Authors:** Jennifer Martinez, Xiaopei Huang, Yiping Yang

**Affiliations:** 1 Department of Immunology, Duke University Medical Center, Durham, North Carolina, United States of America; 2 Department of Medicine, Duke University Medical Center, Durham, North Carolina, United States of America; Fox Chase Cancer Center, United States of America

## Abstract

Natural killer (NK) cells play an essential role in innate immune control of poxviral infections in vivo. However, the mechanism(s) underlying NK cell activation and function in response to poxviruses remains poorly understood. In a mouse model of infection with vaccinia virus (VV), the most studied member of the poxvirus family, we identified that the Toll-like receptor (TLR) 2-myeloid differentiating factor 88 (MyD88) pathway was critical for the activation of NK cells and the control of VV infection in vivo. We further showed that TLR2 signaling on NK cells, but not on accessory cells such as dendritic cells (DCs), was necessary for NK cell activation and that this intrinsic TLR2-MyD88 signaling pathway was required for NK cell activation and played a critical role in the control of VV infection in vivo. In addition, we showed that the activating receptor NKG2D was also important for efficient NK activation and function, as well as recognition of VV-infected targets. We further demonstrated that VV could directly activate NK cells via TLR2 in the presence of cytokines in vitro and TLR2-MyD88-dependent activation of NK cells by VV was mediated through the phosphatidylinositol 3-kinase (PI3K)-extracellular signal-regulated kinase (ERK) pathway. Taken together, these results represent the first evidence that intrinsic TLR signaling is critical for NK cell activation and function in the control of a viral infection in vivo, indicate that multiple pathways are required for efficient NK cell activation and function in response to VV infection, and may provide important insights into the design of effective strategies to combat poxviral infections.

## Author Summary

NK cells are an important component of innate immunity in fighting against poxviral infections in vivo. However, how NK cells are activated and exert their function in controlling poxviruses remains poorly understood. In this paper, we found that VV, the most studied member of the poxvirus family, could directly activate TLR2 on NK cells and that the direct TLR2 stimulation was critical for NK cell activation and function in the control of VV infection in vivo. We further showed that TLR2-dependent NK cell activation by VV was mediated through the PI3K-ERK pathway. In addition, we demonstrated that the activating receptor NKG2D was also required for efficient NK cell activation and function. Collectively, these results represent the first evidence that direct TLR signaling is crucial to NK cell activation and function in the control of a viral infection in vivo, indicate that multiple pathways are required for efficient NK cell activation, and may provide important insights into the design of effective strategies to combat poxviral infections.

## Introduction

Vaccinia virus (VV) is a member of the *Orthopoxvirus* genus of the Poxviridae family, including smallpox (variola) virus, monkeypox virus, cowpox virus, and mousepox (ectromelia) virus. It has a large and complex, double-stranded DNA genome, measuring about 200 Kb, that encodes most of the genes required for cytoplasmic replication of the virus [1]. VV is the most studied member of the poxvirus family and is the live vaccine responsible for successful elimination of smallpox in the late 1970s [2]. This triumph is now being threatened by bioterrorists deliberately reintroducing smallpox, against which vaccination is no longer routine [3]–[5]. Thus, widespread public vaccination is being considered to counter this potential threat. However, the currently used live VV vaccine is associated with a relatively high incidence of severe adverse events, particularly in individuals with eczema and immunodeficiency [6]–[9]. Therefore, there is an imminent need to explore new and safe approaches to control, not only the actual smallpox infection, but also the potential complications from smallpox vaccination with the live VV.

Critical for the development of novel strategies is a better understanding of the host's defense mechanism(s) against poxviruses in vivo. Recent advances have shown that recovery from viral infections depends on the host's ability to mount effective innate immune responses. NK cells represent an important component of the innate immune system and play a critical role in innate immune defense against various viral infections in vivo [10],[11]. Clinically, individuals who are NK cell-deficient suffer from severe, recurrent viral infections [12]. NK cells are also crucial in the control of poxviruses. Upon poxviral infection, NK cells are activated, expand and accumulate at the site of infection, and these activated NK cells are important for the clearance of the infection [13]–[16]. Activation of NK cells is tightly controlled by both inhibitory and activating receptors [17]. Previous studies have shown that upon murine CMV (MCMV) infection, NK cell activation is mediated by the NK cell activating receptor, Ly49H, which specifically recognizes the m157 gene product of MCMV expressed on the surface of infected cells [18],[19]. Similarly, recognition of influenza virus hemagglutinin on virus-infected cells by another activating receptor, NKp46, activates lysis by human NK cells [20], and the murine NKp46 equivalent, NCR1, is required for protection against lethal influenza infection [21]. In addition, the NKG2D activating receptor has been shown to recognize host stress proteins induced upon viral infections including human CMV and MCMV infections [22],[23].

How NK cells are activated upon poxviral infection remains poorly understood. It is known that Ly49H is not involved in the control of VV in mice [24],[25]. Studies in vitro have shown that recognition of VV-infected cells by human NK cells is, in part, mediated by NKp30, NKp44 and NKp46, but not NKG2D [26]. On the other hand, recent studies have suggested that NKG2D is partially involved in NK cell-mediated control of mousepox virus [27]. Thus, mechanisms underlying NK cell responses upon poxviral infection remain largely undefined. In a murine model of VV infection, we have previously shown that VV activates the innate immune system through both the Toll-like receptor (TLR)-dependent and -independent pathways [28]. The TLR pathway is mediated by TLR2 and dependent on MyD88, leading to production of pro-inflammatory cytokines, IL-6, IL-1, and IL-12, whereas activation of the TLR-independent pathway results in the secretion of type I IFN. More importantly, both TLR2 and TLR-independent pathways are required for innate immune control of VV infection [28]. We have shown that the critical role of the TLR-independent pathway in innate immune control of VV is mediated by type I IFN, which acts directly on NK cells to regulate their activation and function [16]. However, how the TLR2-MyD88 pathway contributes to innate immune control of VV remains unclear.

In this report, we provided evidence that the TLR2-MyD88 pathway is also critical for NK cell activation and function in response to VV infection in vivo. This was independent of TLR2-induced production of pro-inflammatory cytokines. We showed that direct TLR2 signaling on NK cells was necessary for efficient NK cell activation upon stimulation with VV and played a critical role in VV control in vivo. In addition, we showed that the NKG2D pathway was also required for efficient NK activation and function, as well as recognition of VV-infected targets. Furthermore, we demonstrated that that VV could directly activate NK cells via TLR2 in the presence of cytokines in vitro and TLR2-dependent activation of NK cells by VV was mediated through the MyD88-PI3K-ERK pathway. Collectively, these results suggest that efficient NK cell activation depends on multiple pathways in response to VV infection and that direct TLR2 activation on NK cells is critical for their function and could lead to the development of novel strategies to combat poxviral infection.

## Results

### NK cell activation and function upon VV infection depends on the TLR2-MyD88 pathway

**Figure 1:**
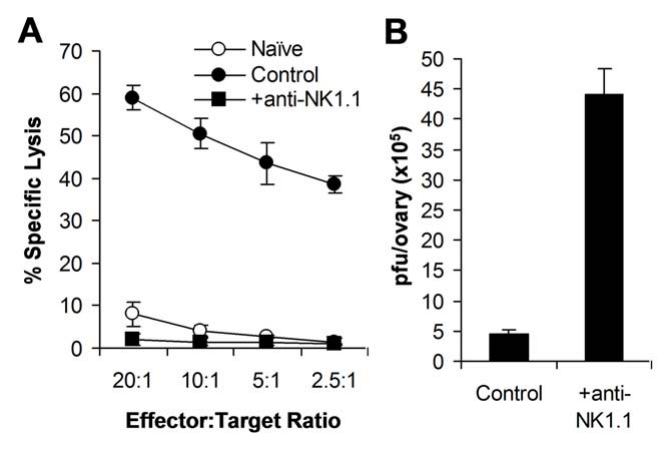
NK cells are required for the control of VV infection in vivo. Female C57BL/6 mice were depleted of NK cells with anti-NK1.1 antibodies (25 µg) on days −3 and 0 (+anti-NK1.1) or left untreated (Control), followed by infection with VV on day 0. (A) 48 h after infection, splenocytes were assayed for NK lytic activity on YAC-1 cells for 4 hr at different effector∶target ratios. Naïve splenocytes (Naïve) were used for comparison. The percentage of specific lysis is shown. (B) The ovaries were assayed for viral load. Data represents viral titer ± SD as pfu per ovary.

Previous studies have shown that NK cells are critical for the control of VV infection in vivo using antibodies to asialo GM1, but not NK1.1 [13]–[16]. Anti-asialo GM1 depletes NK cells and some T cells, but not NKT cells, whereas anti-NK1.1 depletes NK and NKT cells, but not T cells. Thus, to further confirm the role of NK cells in VV control in vivo, we depleted mice of NK cells with anti-NK1.1 antibodies (PK136), followed by infection with VV intraperitoneally. Here we showed that mice depleted of NK cells with anti-NK1.1 also had a defect in NK cell lytic activity (Figure 1A) and a significantly (*p* < 0.001) higher viral titer than the control mice (Figure 1B).

**Figure 2:**
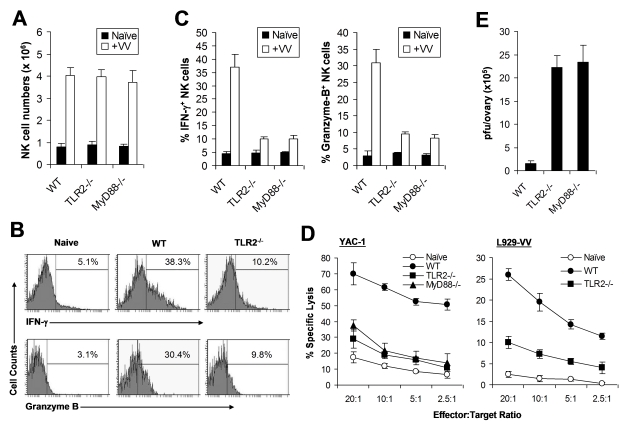
NK cell activation and function in response to VV in vivo requires an intact TLR2-MyD88 pathway. (A–C) Wild-type (WT), TLR2^−/−^, or MyD88^−/−^ mice were infected with VV (+VV) or left uninfected (Naïve). 48 h later, splenocytes were assayed for total NK cell numbers, intracellular IFN-γ and Granzyme B production by NK cells, as well as NK cell lytic assay. (A) The mean numbers ± SD of total DX5^+^CD3^−^ NK cells per spleen are indicated (n  =  6 per group). (B) FACS plots of intracellular IFN-γ and Granzyme B production by NK cells with the percentage of IFN-γ or Granzyme B positive cells among DX5^+^CD3^−^ NK cells indicated. (C) The mean percentage ± SD of IFN-γ or Granzyme B positive cells among DX5^+^CD3^−^ cells is indicated (n  =  6 per group). (D) 48 h after infection, splenocytes were harvested and NK cell lytic activity was assayed on YAC-1 cells or VV-infected L929 cells (L929-VV) for 4 hr at different effector∶target ratios. Naïve splenocytes (Naïve) were used as a control. The mean percentage ± SD of specific lysis is indicated (n  =  6 per group). (E) The ovaries of female mice were harvested for measurement of viral load. Data represents the mean viral titer ± SD as plaque-forming units (pfu) per ovary (n  =  6 per group). Data shown is representative of three independent experiments.

We next investigated how the TLR2-MyD88 pathway contributed to innate immune control of VV. We hypothesized that TLR2-dependent control of VV infection was also mediated through the regulation of NK cell activation. To test this hypothesis, WT, TLR2-deficient (TLR2^−/−^), or MyD88^−/−^ mice were infected with VV intraperitoneally, and splenic NK cells were analyzed for their activation and function. We first showed that splenic NK cell numbers (Figure 2A) and phenotypic markers (Figure S1) from naïve TLR2^−/−^ or MyD88^−/−^ mice were similar to those from WT mice. 48 h after infection, at which time splenic NK cell activation peaked upon VV infection as shown previously [16], splenic NK cells from WT mice produced significantly (*p* <0.001) higher amounts of effector molecules such as IFN-γ and granzyme B, compared to the uninfected naïve control (Figure 2B, C), indicating that these NK cells are activated upon VV infection in vivo. In addition, these NK cells were functionally active, as they demonstrated lytic activities on NK-sensitive YAC-1 cells or VV-infected L929 cells (Figure 2D). In contrast, the production of IFN-γ and granzyme B by splenic NK cells from TLR2^−/−^ or MyD88^−/−^ mice was significantly (*p* <0.001) reduced, compared to the WT controls (Figure 2B, C). Furthermore, splenic NK cells from VV-infected TLR2^−/−^ or MyD88^−/−^ mice displayed drastically diminished lytic activities (Figure 2D), leading to a significant (*p* <0.001) increase in viral load in the ovary (Figure 2E). Collectively, these results indicate that NK cell activation and their effector function in response to VV infection is critically dependent on the TLR2-MyD88 pathway in vivo. However, the expansion of NK cells upon VV infection appeared unchanged in the absence of TLR2 signaling (Figure 2A).

### NK cell activation is independent of TLR2-induced pro-inflammatory cytokines

**Figure 3:**
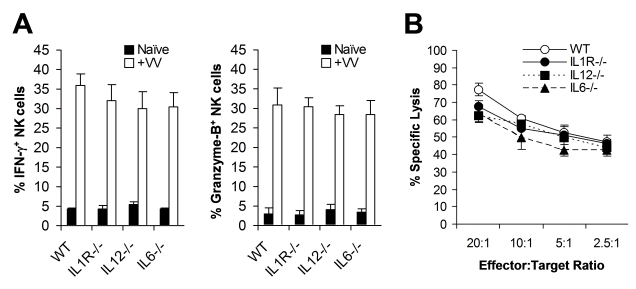
NK cell activation upon VV infection is independent of TLR2-induced production of pro-inflammatory cytokines. (A) WT, IL1R^−/−^, IL12^−/−^, and IL6^−/−^ mice were infected with VV (+VV) or left uninfected (Naïve), and splenocytes were assayed for intracellular IFN-γ and Granzyme B production by NK cells 48 hr later. The percentage ± SD of IFN-γ or Granzyme B positive cells among DX5^+^CD3^−^ cells is indicated (n  =  6). (B) Splenocytes were harvested 48 hr after infection with VV, and NK lytic activity was assayed on YAC-1 cells for 4 hr at different effector∶target ratios. The mean percentage ± SD of specific lysis is indicated (n  =  6). Data shown is representative of three independent experiments.

What then is responsible for TLR2-dependent NK cell activation and function in response to VV infection in vivo? We have previously shown that activation of TLR2 on DCs and macrophages by VV leads to the production of pro-inflammatory cytokines including IL-6, IL-1, and IL-12 [28]. Since IL-12 and IL-1 have been implicated in regulating NK cell activation and function in other models of viral infection [10],[11], we investigated whether the TLR2-dependent NK cell response to VV infection was due to TLR2-induced secretion of pro-inflammatory cytokines. WT, IL-1 receptor-deficient (IL-1R^−/−^), IL-12^−/−^, or IL-6^−/−^ mice were infected with VV intraperitoneally, and NK cells were analyzed 48 h later. No significant differences (*p* >0.05) were observed in the production of IFN-γ or granzyme B by splenic NK cells from IL-1R^−/−^, IL-12^−/−^, or IL-6^−/−^ mice, compared to the WT controls (Figure 3A). In addition, splenic NK cells from IL-1R^−/−^, IL-12^−/−^, or IL-6^−/−^ mice displayed similar levels of lytic activities on YAC-1 targets compared to WT mice (Figure 3B), and no differences in viral load were observed among all mice (data not shown). These results suggest that TLR2-induced IL-12, IL-1 and IL-6 are not critical for NK cell activation and effector function in response to VV infection in vivo.

### TLR2 signaling on NK cells, but not on DCs, is required for NK cell activation

**Figure 4:**
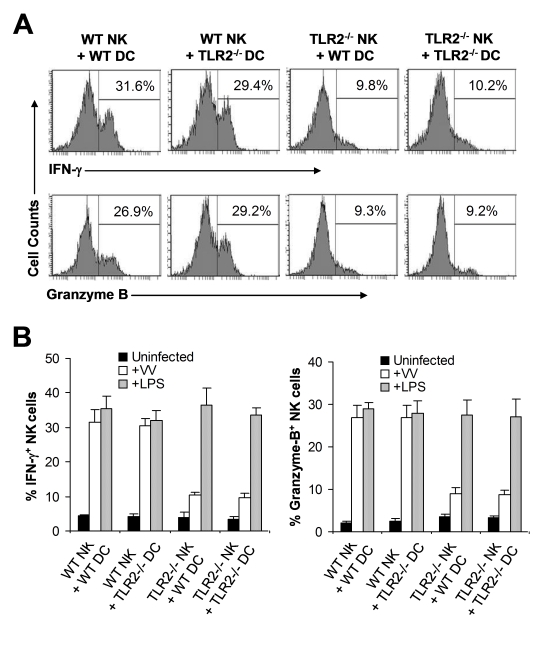
TLR2 signaling on NK cells, but not on DCs, is required for NK cell activation to VV in vitro. WT or TLR2^−/−^ DX5^+^CD3^−^ NK cells were co-cultured with WT or TLR2^−/−^ CD11c^+^ DCs and stimulated with VV (+VV), LPS (+LPS) or left uninfected (Uninfected). 48 hr after infection, NK cells were assayed for intracellular IFN-γ and Granzyme B. (A) The percentage of IFN-γ or Granzyme B positive cells among DX5^+^CD3^−^ cells is indicated. (B) The mean percentage ± SD of IFN-γ or Granzyme B positive cells among DX5^+^CD3^−^ cells is shown. Data shown is representative of two independent experiments.

The observation that NK cell activation and function upon VV infection in vivo is independent of TLR2-induced IL-1, IL-6, and IL-12 production may not completely rule out the role of TLR2 signaling on accessory cells, such as DCs, in NK cell activation as VV could stimulate TLR2 on DCs to secrete other cytokines or activate other pathways that may be critical for NK cell activation. To address this question, we utilized an in vitro DC-NK cell co-culture system as described [16]. DX5^+^CD3^−^ splenic NK cells were isolated by FACS sorting with a purity of >98% (Figure S2). Purified WT or TLR2^−/−^ NK cells were co-cultured in vitro with WT or TLR2^−/−^ CD11c^+^ DCs, followed by infection with VV. 48 h later, NK cells were analyzed for the production of IFN-γ and granzyme B. Our data showed that similar amounts of IFN-γ and granzyme B were produced by WT NK cells when cultured with TLR2^−/−^ DCs to those with WT DCs (Figure 4A, B). In addition, WT NK cells co-cultured with TLR2^−/−^ DCs displayed similar levels of lytic activity on YAC-1 targets to those with WT DCs (data not shown). These results suggest NK cell activation is independent of TLR2 signaling on DCs in response to VV infection. In contrast, NK cell activation was severely compromised when TLR2^−/−^ NK cells were used for stimulation (Figure 4A, B), indicating that direct TLR2 signaling on NK cells is required for their activation upon VV infection. The lack of IFN-γ and granzyme B production by TLR2^−/−^ NK cells was not due to their inherent inability to be activated as TLR2^−/−^ NK cells stimulated with TLR4 ligand, LPS, produced similar levels of IFN-γ and granzyme B compared to the WT NK cells (Figure 4B). Taken together, these results suggest that TLR2 signaling on NK cells, but not accessory DCs, is required for the activation of NK cells by VV.

### Direct TLR2-MyD88 signaling is required for NK cell activation and function in response to VV infection in vivo

**Figure 5:**
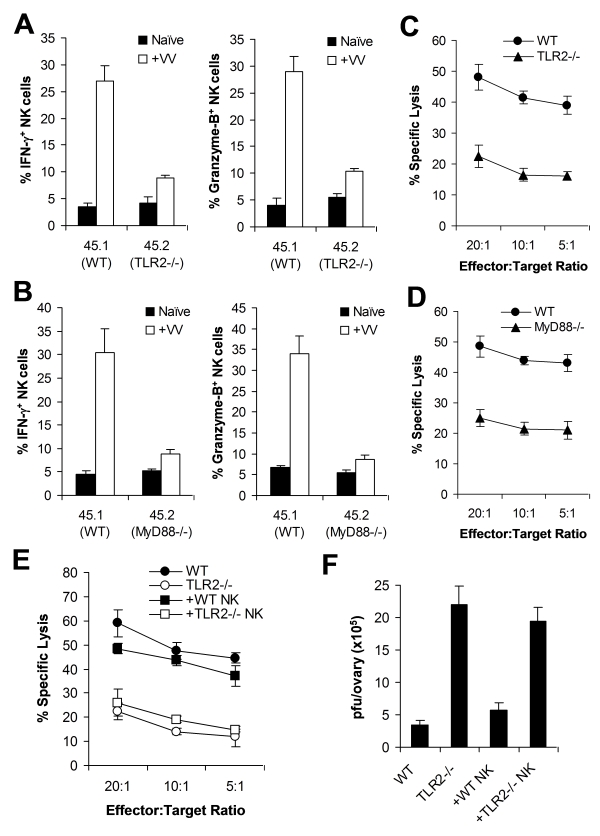
Direct TLR2-MyD88 signaling is required for NK cell priming in response to VV in vivo. (A–D) Bone marrow chimeric mice were generated by reconstituting irradiated CD45.1^+^ WT mice with bone marrow cells from CD45.1^+^ WT and CD45.2^+^ TLR2^−/−^ (A, C) or MyD88^−/−^ (B, D) mice at a 1∶1 ratio. 6–8 weeks after reconstitution, chimeric mice were infected with VV (+VV) or left uninfected (Naïve). 48 hr later, splenocytes were assayed for intracellular IFN-γ and Granzyme B production by NK cells. The mean percentage ± SD of IFN-γ or Granzyme B positive cells among CD45.1^+^ or CD45.2^+^ DX5^+^CD3^−^ cells is indicated (n  =  4 per group) (A, B). Splenocytes were sorted into CD45.1^+^ or CD45.2^+^ DX5^+^CD3^−^ populations 48 h after VV infection, and NK lytic activity was assayed on YAC-1 cells for 4 hr at different effector∶target ratios. The mean percentages of specific lysis are indicated (n  =  4 per group) (C, D). Data shown is representative of two independent experiments. (E–F) TLR2^−/−^ mice were reconstituted with WT NK cells (+ WT NK) or TLR2^−/−^ NK cells (+ TLR2−/− NK) followed by infection with VV. WT and TLR2^−/−^ mice infected with VV were used as controls. 48 hr later, splenocytes were assayed for NK lytic activity on YAC-1 cells at different effector∶target ratios. The mean percentage of specific lysis is indicated (n  =  4) (E). The ovaries of female mice were harvested for measurement of viral load. Data represents the mean viral titer ± SD as pfu per ovary (n  =  4) (F). Data is representative of two independent experiments.

We next investigated the in vivo relevance of direct TLR2 signaling in NK cell activation and the control of VV infection in vivo. We first evaluated if TLR2-MyD88 signaling on NK cells was critical for NK cell activation in mixed TLR2^−/−^ or MyD88^−/−^, and WT bone marrow chimeric mice. CD45.1^+^ recipient mice were irradiated with 1200 cGy and reconstituted with equal numbers of bone marrow cells harvested from CD45.1^+^ WT and CD45.2^+^ TLR2^−/−^ (or MyD88^−/−^) mice. Mice were allowed to reconstitute their hematopoietic cell populations for 6 to 8 weeks. Mixed chimeric mice were then infected with VV intraperitoneally, or left uninfected as controls. 48 h later, splenocytes were analyzed for activation of WT (CD45.1^+^), and TLR2^−/−^ or MyD88^−/−^ (CD45.2^+^) DX5^+^CD3^−^ NK cells. In addition, WT and TLR2^−/−^ (or MyD88^−/−^) NK cells were purified by FACS and assayed for cytotoxicity on NK-sensitive YAC-1 cells. WT NK cells from VV-infected recipients produced significantly (*p* <0.001) higher amounts of IFN-γ and granzyme B, compared to the uninfected control (Figure 5A, B). However, the production of IFN-γ and granzyme B by TLR2^−/−^ (Figure 5A) or MyD88^−/−^ (Figure 5B) NK cells was significantly (*p* <0.001) reduced compared to the respective WT controls. In addition, TLR2^−/−^ (Figure 5C) or MyD88^−/−^ (Figure 5D) NK cells showed drastically diminished lytic activity compared to the WT NK cells. These results suggest that direct TLR2-MyD88 signaling on NK cells is critical for NK cell activation and their function in vivo.

To further support the role of direct TLR2 signaling on NK cell activation and function, we examined if transfer of WT NK cells into TLR2^−/−^ mice restored NK cell function and resulted in a significant reduction of viral load. DX5^+^CD3^−^ NK cells were purified from the spleens of WT or TLR2^−/−^ mice by FACS sorting. Purified WT or TLR2^−/−^ NK cells were then transferred into TLR2^−/−^ mice intravenously, which were subsequently infected with VV intraperitoneally. After 48 h, the spleens and ovaries from these recipient mice were analyzed for NK cell activation and viral titer. In TLR2^−/−^ mice reconstituted with WT NK cells, the production of IFN-γ and granzyme B by splenic NK cells neared that in WT mice (data not shown). Furthermore, splenic NK cells harvested from TLR2^−/−^ mice reconstituted with WT, but not TLR2^−/−^, NK cells were capable of lysing YAC-1 targets to a level equivalent to that of WT mice (Figure 5E). When VV titer in the ovaries was assessed, TLR2^−/−^ mice reconstituted with WT NK cells were able to clear VV in vivo similarly to WT mice, whereas TLR2^−/−^ mice or TLR2^−/−^ mice reconstituted with TLR2^−/−^ NK cells failed to clear the virus (Figure 5F). Taken together, these data support the conclusion that intrinsic TLR2-MyD88 signaling on NK cells is required for NK cell activation and function in response to VV infection in vivo.

### Efficient NK cell activation and function upon VV infection is also dependent on the NKG2D pathway

Despite a critical role for the direct TLR2 signaling in NK cell activation, the production of IFN-γ and granzyme B by TLR2^−/−^ NK cells in response to VV infection was 2–3 folds above the background (Figures 2 & 4), suggesting NK cell activation is not completely abolished in the absence of TLR2 signaling. Indeed, depletion of NK cells with anti-NK1.1 (Figure S3A) or anti-asialo GM1 (data not shown) in TLR2^−/−^ mice led to an increase viral titer over the control TLR2^−/−^ mice (Figure S3A). Furthermore, transfer of TLR2^−/−^ NK cells into NK cell-depleted WT mice also resulted in a small reduction in viral load (Figure S3B). Collectively, these data suggest the existence of a TLR2-independent pathway for efficient NK cell activation.

**Figure 6:**
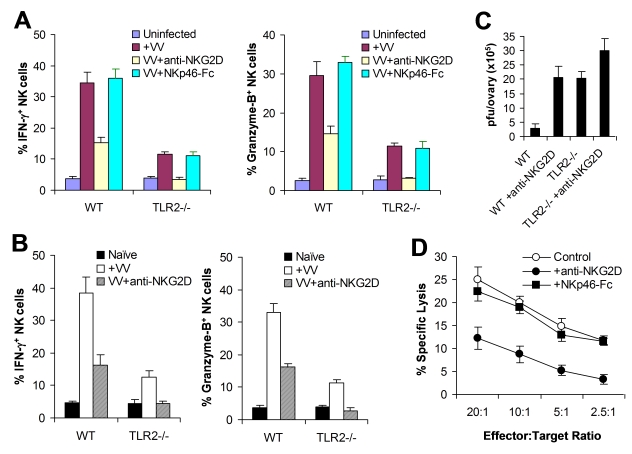
The NKG2D pathway is required for efficient NK activation and function in response to VV infection. (A) WT or TLR2^−/−^ DX5^+^CD3^−^ NK cells were co-cultured with WT CD11c^+^ DCs and stimulated with VV alone (+VV), VV in the presence of anti-NKG2D (VV+anti-NKG2D) or NKp46-Fc chimera (VV+NKp46-Fc), or left uninfected (Uninfected). 24 hr later, NK cells were assayed for intracellular IFN-γ and Granzyme B. The mean percentage ± SD of IFN-γ or Granzyme B positive cells among DX5^+^CD3^−^ cells is shown. (B–C) WT or TLR2^−/−^ mice were infected with VV (+VV) or left uninfected (Naïve). Some mice were pre-treated with anti-NKG2D antibodies 24 and 6 h prior to infection with VV (VV+anti-NKG2D). 48 h after infection, splenic NK cells were analyzed for IFN-γ and Granzyme B production. The mean percentage ± SD of IFN-γ or Granzyme B positive cells among DX5^+^CD3^−^ cells is indicated (n  =  4 per group) (B). The ovaries of female mice were harvested for measurement of viral load. Data represents the mean viral titer ± SD as pfu per ovary (n  =  4 per group) (C). (D) 48 h after infection, splenocytes from WT mice were assayed for NK cell lytic activity on VV-infected L929 cells in the presence of anti-NKG2D antibodies (+anti-NKG2D) or NKp46-Fc chimera (+NKp46-Fc), for 4 hr at different effector∶target ratios. The mean percentage ± SD of specific lysis is indicated (n  =  4 per group). Data shown is representative of two independent experiments.

Previous studies have shown that NKG2D is partially involved in NK cell-mediated control of mousepox virus in vivo [27], and that recognition of VV-infected cells by human NK cells is, in part, mediated by natural cytotoxicity receptors, NKp30, NKp44 and NKp46 [26]. Among natural cytotoxicity receptors, only NKp46 is expressed in mice. Thus, we investigated whether NKG2D or NKp46 contributed to TLR2-independent NK cell activation in response to VV infection. We first tested this in vitro with the NK-DC co-culture system. Purified NK cells from WT or TLR2^−/−^ mice were co-cultured in vitro with WT CD11c^+^ DCs in the presence of a blocking anti-NKG2D antibody or a blocking NKp46-Fc fusion protein, followed by infection with VV, the activation of NK cells was analyzed 24 h later. The production of IFN-γ and granzyme B by WT NK cells was significantly (*p* <0.01) decreased in the presence of anti-NKG2D compared to the control without anti-NKG2D (Figure 6A). In addition, the production of IFN-γ and granzyme B by TLR2^−/−^ NK cells was completely abolished with the NKG2D blocking (Figure 6A). However, blocking with NKp46-Fc had no effect on activation of WT or TLR2^−/−^ NK cells (Figure 6A). These results indicate that NKG2D, but not NKp46, is also involved in NK cell activation upon VV infection.

We next examined the role of NKG2D in NK cell activation and function in vivo. WT or TLR2^−/−^ mice were injected with the blocking anti-NKG2D antibody intravenously 24 h and 6 h prior to infection with VV intraperitoneally, and splenic NK cells were analyzed for their activation and function 48 h after infection. In WT mice, NK cells produced significantly (*p* <0.01) less amounts of IFN-γ and granzyme B in the presence of NKG2D blocking, compared to the control without NKG2D blocking (Figure 6B), leading to a significant (*p* <0.001) increase in viral load in the ovaries (Figure 6C). Furthermore, in TLR2^−/−^ mice, NK cell activation was completely abolished with NKG2D blocking (Figure 6B), leading to a further increase in viral load in the ovaries (Figure 6C). These data indicate that the NKG2D pathway is also important in the activation of NK cells and the control of VV infection in vivo.

To address whether NKG2D or NKp46 is involved in the NK cell recognition of VV-infected targets, NK cells harvested from VV-infected WT mice were assayed for their lytic activities on VV-infected L929 targets in vitro in the presence of NKG2D or NKp46 blocking. A significant reduction in cytolytic activities was observed with the NKG2D, but not NKp46, blocking (Figure 6D), suggesting NKG2D is also critical for the recognition of VV-infected cells.

Collectively, these results suggest that the TLR2-independent NKG2D pathway is also required for efficient NK cell activation, recognition of VV-infected targets and VV control in vivo.

### VV can directly activate NK cells via TLR2 in the presence of cytokines in vitro

**Figure 7:**
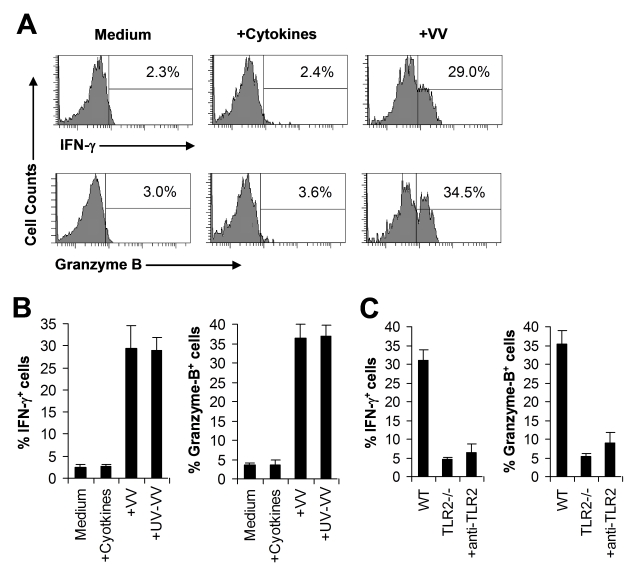
VV activates NK cells directly via TLR2 in vitro. (A–B) DX5^+^CD3^−^ NK cells were cultured in the medium alone (Medium) or medium supplemented with IL-2 and IFN-α (+Cytokines), or infected with live VV (+VV) or UV-inactivated VV (+UV-VV) in the presence of IL-2 and IFN-α. 48 h later, NK cells were assayed for intracellular IFN-γ and Granzyme B. The percentage of IFN-γ or Granzyme B positive cells among DX5^+^CD3^−^ cells is indicated (A). The mean percentage ± SD of IFN-γ or Granzyme B positive cells among DX5^+^CD3^−^ cells is shown (B). (C) DX5^+^CD3^−^ NK cells from WT or TLR2^−/−^ mice were stimulated with VV for 48 h and assayed for intracellular IFN-γ and Granzyme B. Some WT NK cells were stimulated with VV in the presence of a blocking TLR2 antibody (+anti-TLR2). The percentage ± SD of IFN-γ or Granzyme B positive cells among DX5^+^CD3^−^ cells is shown. Data shown is representative of three independent experiments.

The observation that direct TLR2 signaling on NK cells is required for NK cell activation and function in response to VV infection suggested that VV might directly activate NK cells via TLR2. To address this question, an accessory cell-free NK culture system is required. However, an accessory cell-free system would lack NKG2D stimulation as NKG2D ligands are usually expressed by accessory cells upon viral infection. To compensate for lack of NKG2D stimulation, we established an accessory cell-free NK culture system in the presence of low dose IL-2 and IFN-α as they have been used for the activation of NK cells in vitro. FACS-purified splenic DX5^+^CD3^−^ NK cells were stimulated with VV and assayed for NK cell activation 48 h later. No significant IFN-γ or granzyme B production was detected in the presence of IL-2 and IFN-α compared to the medium control (Figure 7A, B), suggesting that IL-2 and IFN-α alone do not activate NK cells. However, addition of VV stimulated NK cells to produce significantly (*p* <0.001) higher levels of IFN-γ and granzyme B (Figure 7A, B). These results indicate that VV can directly activate NK cells in the presence of cytokines. Similar results were obtained when UV-inactivated VV was used for stimulation (Figure 7B), suggesting that NK cell stimulation by VV is independent of newly synthesized viral products after infection.

We next investigated whether VV activated NK cells via TLR2. It has been shown NK cells express multiple TLRs [29]–[31]. Indeed, we showed here that TLR2, TLR4, TLR8, and TLR9 were expressed at the RNA level in NK cells (Figure S4A). Furthermore, we found that TLR2 protein is expressed on the surface of freshly isolated NK cells by FACS and that the level of TLR2 expression remained constant after incubation with cytokines or stimulation with VV (Figure S4B). When purified NK cell from TLR2^−/−^ or TLR9^−/−^ mice were stimulated with VV, the production of IFN-γ and granzyme B by TLR2^−/−^ (Figure 7C), but not TLR9^−/−^ (data not shown), NK cells was significantly (*p* <0.001) reduced, compared to WT controls. To more directly confirm that VV activated NK cells via TLR2, WT NK cells were pre-treated with a blocking TLR2 antibody and stimulated with VV. Our result showed that TLR2 blocking led to a significant reduction in IFN-γ and granzyme B production by WT NK cells (Figure 7C). Taken together, these data indicate that VV can directly activate NK cells via TLR2.

### TLR2-MyD88-dependent NK cell activation by VV is mediated by the PI3K-ERK pathway

**Figure 8:**
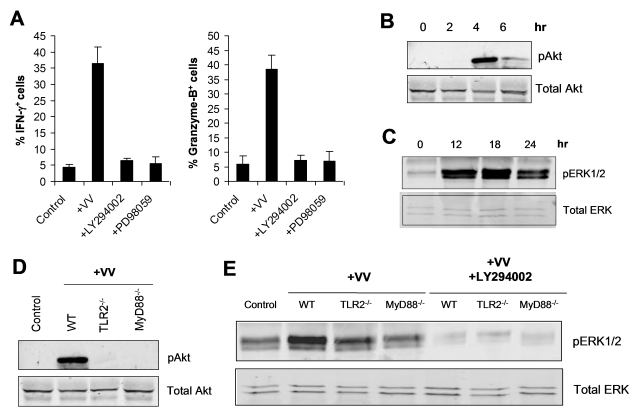
TLR2-dependent activation of NK cells by VV is mediated by PI3K and ERK. (A) DX5^+^CD3^−^ NK cells were infected with VV (+VV), VV in the presence of the PI3K inhibitor, LY294002 (+LY294002) or the ERK inhibitor, PD98059 (+PD98059) in vitro. 48 h after infection, NK cells were assayed for intracellular IFN-γ and Granzyme B. The mean percentage ± SD of IFN-γ or Granzyme B positive cells among DX5^+^CD3^−^ cells is shown. Data shown is representative of three independent experiments. (B–C) DX5^+^CD3^−^ NK cells were stimulated in vitro with VV for the indicated time periods. After stimulation for 2, 4, and 6 h, NK cells were removed from culture and total cell lysates were collected for Western blot analysis of phosphorylated Akt (pAkt) as well as total Akt (Total Akt), which served as a loading control (B). After stimulation for 12, 18, and 24 h, NK cells were removed from culture and total cell lysates were collected for Western blot analysis of phosphorylated ERK1/2 (pERK1/2) as well as total ERK (Total ERK) (C). (D) WT, TLR2^−/−^, and MyD88^−/−^ DX5^+^CD3^−^ NK cells were stimulated in vitro with VV (+VV), or left unstimulated (Control) for 4 h. NK cells were removed from culture and total cell lysates were collected for Western blot analysis of phosphorylated Akt (pAkt) as well as total Akt (Total Akt). (E) WT, TLR2^−/−^, and MyD88^−/−^ DX5^+^CD3^−^ NK cells were stimulated in vitro with VV (+VV), VV and the PI3K inhibitor LY294002 (+LY294002), or left unstimulated (Control) for 18 h. NK cells were removed from culture and total cell lysates were collected for Western blot analysis of phosphorylated ERK1/2 (pERK1/2) as well as total ERK (Total ERK). Data shown is a representative blot of five independent experiments.

How does stimulation of TLR2-Myd88 pathway on NK cells by VV lead to NK cell activation? Recent studies have shown that in T cells, MyD88 can interact with PI3K and activate the PI3K pathway, leading to enhanced T cell activation and survival upon TLR stimulation [32], and PI3K is the common signaling mediator downstream of activating NK receptors, such as ITAM-bearing NK receptors and NKG2D [17]. Furthermore, the PI3K-ERK pathway has been shown to play an important role in NK cell activation and cytotoxicity [33],[34]. Thus, we hypothesized that TLR2-MyD88 signaling on NK cells activated the downstream PI3K-ERK pathway, leading to activation of NK cells upon VV infection. To test this hypothesis, we first examined if activation of NK cells by VV was mediated by PI3K and ERK. FACS-purified NK cells were stimulated with VV in the presence of the PI3K inhibitor, LY294002 or the ERK1/2 inhibitor, PD98059 and analyzed for their activation 48 h later. Indeed, the production of IFN-γ and granzyme B by NK cells was significantly (*p* <0.001) reduced in the presence of LY294002 or PD98059, compared to the control without inhibitors (Figure 8A), suggesting that both PI3K and ERK are involved in NK cell activation by VV.

We next tested whether stimulation of NK cells with VV led to the activation of both PI3K and ERK in a TLR2-MyD88-dependent manner. Stimulation of NK cells with VV resulted in the activation of PI3K, evidenced by phosphorylation of Akt, an immediate downstream target of PI3K (indicative of PI3K activation), peaked at 4 h after stimulation (Figure 8B). Similarly, ERK was also activated upon stimulation with VV, peaked at 18 h after the infection (Figure 8C). The phosphorylation of both PI3K and ERK was critically dependent on an intact TLR2-MyD88 pathway as the activation of Akt (Figure 8D) and ERK (Figure 8E) was greatly diminished in TLR2^−/−^ or MyD88^−/−^ NK cells stimulated with VV, compared to that in the WT controls. Furthermore, ERK phosphorylation was severely compromised in the presence of LY294002 (Figure 8E), confirming that ERK is downstream of PI3K. Taken together, these data indicate that TLR2-MyD88-dependent NK cell activation by VV is mediated by the PI3K-ERK pathway.

## Discussion

NK cells play a critical role in the control of poxviral infection in vivo [13]–[16]. We have previously shown that type I IFN, induced upon VV infection, acts directly on NK cells to regulate their activation and function [16]. However, the mechanism(s) by which NK cells are activated and function in response to poxviral infection remains poorly understood. In this study, we provided evidence that activation of the TLR2-MyD88 innate immune pathway is critical for NK cell activation and function in response to VV infection in vivo. Since VV activates TLR2 on accessory cells, such as DCs and macrophages, to secrete pro-inflammatory cytokines IL-6, IL-1, and IL-12 [28], this dependence on TLR2 pathway could be mediated indirectly through IL-1 and IL-12, as these cytokines have been shown to play a role in NK cell activation [10],[11]. However, our data demonstrates that NK cell activation and function is independent of TLR2-induced IL-1 and IL-12 in vivo and that TLR2 signaling on NK cells, but not on DCs, is necessary for NK cell activation upon stimulation with VV in vitro. This data suggest a role for direct TLR2 stimulation on NK cells by VV in NK cell activation and function.

Indeed, the observations that TLR2 is expressed on NK cells, that VV can directly activate NK cells via TLR2 in the absence of accessory cells, and that intrinsic TLR2-MyD88 signaling is required for NK cell activation and function in the control of VV in vivo, support a critical role for direct TLR2 stimulation on NK cells for their activation and function. Although it has been shown that TLR3, TLR7, TLR8, and TLR9 are also expressed on human NK cells and that ligands for these TLRs can activate human NK cells in vitro [29]–[31], our observations reveal for the first time that direct TLR stimulation is critical for NK cell activation and function in the control of viral infection in vivo. Thus, in the context of sensing VV, TLR2 may represent a novel class of NK cell activating receptor that is distinct from NKG2D, which detects stress-induced ligands [10],[11], as well as Ly49H and NKp46, both of which recognize pathogen-derived products expressed on infected cells [18]–[21].

Although NK cell activation in response to MCMV is mediated by NK cell activation receptor, Ly49H, which specifically recognizes the m157 gene product of MCMV [18],[19], Ly49H is not involved in the control of VV in mice [24],[25]. The role of other NK activating receptors, such as NKp46 [20],[21] and NKG2D [22],[23], in NK cell activation and recognition of poxviruses remain controversial [26],[27], and their precise role in vivo remains to be defined. Here we showed that NK cell activation, recognition of VV-infected target cells by NK cells and NK cell-mediated control of VV infection in vivo are also dependent on NKG2D, but not NKp46. Thus, multiple pathways are required for efficient activation of NK cells as well as their function in the control of VV infection in vivo. Future work is required to delineate how TLR2-dependent and –independent pathways cooperate to achieve efficient NK cell activation in the control of VV infection in vivo.

We have further shown that direct TLR2-MyD88 signaling on NK cells activates the downstream PI3K-ERK pathway, leading to activation of NK cells upon VV infection. This is in line with previous observations that PI3K is the common signaling mediator downstream of activating NK receptors, such as ITAM-bearing NK receptors and NKG2D [17]. Furthermore, the PI3K-ERK pathway has been shown to play an important role in NK cell activation and cytotoxicity [33],[34]. How does stimulation of TLR2 on NK cells lead to activation of the PI3K-ERK pathway? Recent studies have shown that in CD4 T cells, MyD88 can directly interact with PI3K and activate the PI3K pathway, leading to enhanced T cell activation and survival upon TLR stimulation [32]. Similarly, we have recently demonstrated that VV can directly stimulate TLR2 on CD8 T cells, which activates the PI3K-Akt pathway, leading to enhanced proliferation and survival of activated CD8 T cells [35]. Whether MyD88 interacts directly or indirectly with PI3K in NK cells requires further investigation.

What component(s) of VV then is responsible for the activation of TLR2 on NK cells? In addition to live VV, UV-VV can also activate NK cells via TLR2, suggesting that the stimulation of TLR2 on NK cells by VV is independent of newly synthesized viral gene products after infection. Although NK cells also express TLR9, the observation that NK cell activation by VV is independent of TLR9 (data not shown), which recognizes CpG DNA ligand, suggests that VV DNA is unlikely the ligand to activate NK cells. These results, coupled with our previous observation that both live VV and UV-VV can induce TLR2-dependent production of pro-inflammatory cytokines peaking at 6 hr after infection in vivo [28], suggest that the envelope and/or membrane structural components of VV might be responsible for activating TLR2 during virus-cell contact. Indeed, previous studies on other viruses have shown that viral envelope glycoproteins can trigger TLR2 or TLR4 responses [36]–[38]. Specifically, the fusion protein of respiratory syncytial virus (RSV) [36], the envelope protein of mouse mammary tumor virus (MMTV) [38], and hemagglutinin protein of measles virus [37] have been identified as ligands for activating the TLR responsiveness in their respective systems. Thus, it will be important to identify what component(s) of VV triggers NK cell activation in the future. Identification of such a ligand will help us design effective NK cell-based strategies to control poxviral infections in vivo.

In conclusion, we have shown that VV can directly activate NK cells via TLR2 and this intrinsic TLR2-MyD88 signaling pathway was critical for NK cell activation and function in the control of VV infection in vivo. This TLR2-MyD88-dependent NK cell activation by VV was mediated by the PI3K-ERK pathway. Furthermore, there is a TLR2-independent NK cell activation pathway mediated by NKG2D, which is also important for efficient NK cell activation and function in response to VV infection. These results identify for the first time that direct stimulation of TLR on NK cells are critical for their activation and function following a viral infection in vivo and may shed light on the design of effective strategies to combat poxviral infections.

## Materials and Methods

### Mice

CD45.1^+^ and CD45.2^+^ C57BL/6 mice were obtained from The Jackson Laboratory (Bar Harbor, ME). TLR2^−/−^, TLR9^−/−^, and MyD88^−/−^ mice were kindly provided by Shizuo Akira (Osaka University, Osaka, Japan). These mice have been backcrossed onto C57BL/6 background for >9 generations. IL-1R^−/−^, IL-12^−/−^, and IL-6^−/−^ mice on C57BL/6 background were obtained from The Jackson Laboratory. Groups of 6- to 10-wk-old mice were selected for this study. All experiments involving the use of mice were done in accordance with the A-052-09-02 protocol, and were approved by the Animal Care and Use Committee of Duke University.

### Vaccinia virus

The Western Reserve (WR) strain of VV was purchased from American Type Culture Collection (ATCC). VV was grown in TK-143B cells (ATCC) and purified by a 35% sucrose cushion, and the titer was determined by plaque assay on TK-143B cells and stored at −80°C until use as described [28]. UV-inactivation of VV was performed as described with some modifications [28]. Briefly, purified virus was resuspended in 1 µg/ml 8-methoxypsoralen (Sigma) and then exposed to a 365 nm UV light source (UVP model UVGL-25) on ice for 3 min in a 24-well plate. For in vitro NK stimulations, both live VV and UV-inactivated VV were used at MOI of 1. The dose of UV-VV was based on pre-inactivation titer. For in vivo studies, 1×10^7^ pfu of live VV in 0.1 ml of PBS was injected into mice intraperitoneally as described [16].

### Antibodies and flow cytometry

FITC-conjugated anti-IFN-γ (clone XMG1.2), PE-conjugated anti-CD49b/Pan-NK Cells (clone DX5), PE-Cy5-conjugated anti-CD3ε (clone 145-2C11), Biotin-conjugated CD45.2 (clone 104), FITC-conjugated anti-CD69 (clone H1.2F3), FITC-conjugated anti-CD62L (clone MEL-14), and Streptavidin-conjugated APC were purchased from BD Biosciences. FITC-conjugated anti-Granzyme B (clone 16G6) and APC-conjugated anti-KLRG1 (clone 2F1) were purchased from eBioscience. To assess production of IFN-γ and Granzyme B intracellularly, splenocytes were incubated with 100 ng/ml PMA and 250 ng/ml ionomycin and 5 µg/ml Brefeldin A containing Golgi-Plug (BD Biosciences) for 4 hours at 37°C. Intracellular staining was performed as previously described [16]. FACS Canto (BD Biosciences) was used for flow cytometry event collection, which was analyzed using FACS DiVA software (BD Biosciences).

### NK cell cytotoxicity assay

NK cell cytotoxicity assay was performed as previously described [16]. In brief, splenocytes were enriched for DX5^+^ NK cells by positive selection with PE-conjugated anti-DX5 and anti-PE microbeads (Miltenyi Biotec). DX5^+^ cells were then incubated with ^51^Cr-labeled NK sensitive targets, YAC-1 cells (ATCC), at different effector to target cell ratios for 4 hours at 37°C. The specific ^51^Cr release was calculated as (experimental_cpm_−spontaneous_cpm_)/(maximum_cpm_−spontaneous_cpm_)×100. In some experiments, L929 cells (ATCC) infected with VV at an MOI of 20 for 2 hours, were used for targets as described [39].

### Measurement of VV titer

Viral load in the ovaries was measured by plaque-forming assay as described [16],[28]. Briefly, female mice were sacrificed 2 days after infection, and ovaries were harvested and stored at −80°C. Ovaries from individual mice were homogenized and freeze-thawed 3 times. Serial dilutions were performed on confluent TK-143B cells, and viral titers were then determined 2 days later by crystal violet staining.

### In vivo depletion of NK cells

For depletion of NK cells in vivo, mice received 250 µg of anti-asialo GM1 antiserum (Wako Chemicals) or 25 µg of functional grade purified anti-mouse NK1.1 (eBioscience, clone PK136) injected intravenously on day −3 and day 0 of the infection with VV. Before infections, peripheral blood and splenic cells were analyzed to confirm elimination of DX5^+^CD3^−^ NK cells.

For the reconstitution of NK cells after NK cell depletion, mice received only a single dose of anti-NK1.1 or anti-asialo GM1 on day −2, and were reconstituted with purified NK cells (5×10^5^) on day 0 as described [40].

### NK cell-DC co-culture system

DCs were generated from the bone marrow cells as described [28]. Briefly, bone marrow cells were harvested from femurs and tibiae of mice and cultured in the presence of mouse GM-CSF (1,000 U/ml) and IL-4 (500 U/ml) (R & D Systems) for 5 days. GM-CSF and IL-4 were replenished on days 2 and 4. On day 5, CD11c+ DCs were harvested for NK cell stimulation. NK cell-DC co-culture was performed as described [16],[23] with some modifications. Briefly, DX5^+^ NK cells were enriched from splenocytes by positive selection with PE-conjugated anti-DX5 and anti-PE microbeads (Miltenyi Biotec). DX5^+^CD3^−^ NK cells were purified from the enriched DX5^+^ cells via flow cytometry sorting on a FACS DiVA. NK cells (5×10^5^) were co-cultured with CD11c+ DC (2.5×105) at an NK cell∶DC ratio of 2∶1. The co-culture was subsequently stimulated with VV with multiplicity of infection (MOI) of 1, or LPS (100 ng/ml), for 24–48 hours at 37°C.

In some experiments, NK cell cultures were inhibited with 10 µg/ml of functional grade purified anti-mouse NKG2D (eBioscience, clone CX5) or 30 µg/ml of recombinant mouse NKp46/NCR1/Fc Chimera (R & D Systems) as described [27],[41].

### In vivo inhibition of NK cell activity with anti-NKG2D

For inhibition of NKG2D activity in vivo, mice received 100 µg of functional grade purified anti-mouse NKG2D (eBioscience, clone CX5) intravenously 24 and 6 h prior to infection with VV.

### Bone marrow chimeras

CD45.1^+^ C57BL/6 recipient mice were irradiated with 1200 cGy of gamma irradiation and reconstituted with 1×10^6^ of CD45.1^+^ WT and 1×10^6^ of CD45.2^+^ MyD88^−/−^ or TLR2^−/−^ bone marrow cells. Mice were allowed to reconstitute their hematopoietic cell population for 6 to 8 weeks. Chimerism was confirmed prior to experimental use. Mixed chimeric mice were injected with 1×10^7^ pfu VV intraperitoneally, and their spleens were harvested and assayed for NK cell function 48 h later. NK cell cytotoxicity assay was performed after FACS sorting for WT (CD45.1^+^DX5^+^CD3^−^) and MyD88^−/−^ or TLR2^−/−^ (CD45.2^+^DX5^+^CD3^−^) NK cells.

### Reconstitution of NK cells in vivo

DX5^+^CD3^−^ NK cells were purified from splenocytes of naïve mice via FACS sorting. 2×10^5^ DX5^+^CD3^−^ NK cells were administered intravenously to TLR2^/−^ recipients, which were subsequently injected intraperitoneally with 1×10^7^ pfu VV. Analysis was performed 48 h after infection.

### Accessory cell-free NK cell culture

NK cell alone culture was performed as described with some modifications [23]. Briefly, DX5^+^CD3^−^ NK cells were purified from splenocytes of naïve mice via flow cytometry sorting on a FACS DiVA with a purity of >98%. NK cells (1×10^6^) were cultured in the presence of recombinant murine IL-2 (50U/ml) and IFN-α (2000U/ml), and infected with VV with MOI of 2 for 48 h at 37°C. In some experiments, the PI3-K inhibitor, LY294002 (10 µM, Calbiochem), the ERK1/2 inhibitor, PD98059 (50 µM, Calbiochem) or anti-mouse TLR2 antibody (eBioscience, clone T2.5).

### Reverse-transcriptase PCR

Total RNA was isolated from flow cytometry sorted NK cells using Trizol reagent according to manufacturer's instructions. PCR was performed in the presence or absence of reverse transcriptase using template RNA and primers specific for TLR2 (forward 5′TTGCTCCTGCGAACTCCTAT-3′, reverse 5′-CAATGGGAATCCTGCTCACT-3′), TLR3 (forward 5′-CCCCCTTTGAACTCCTCTTC-3′, reverse 5′-TTTCGGCTTCTTTTGATGCT-3′), TLR4 (forward 5′-GCTTTCACCTCTGCCTTCAC-3′, reverse 5′-CGAGGCTTTTCCATCCAATA-3′), TLR7 (forward 5′-GGTATGCCGCCAAATCTAAA-3′, reverse 5′-TTGCAAAGAAAGCGATTGTG-3′), TLR8 (forward 5′-CAAACAACAGCACCCAAATG-3′, reverse 5′-GGGGGCACATAGAAAAGGTT-3′), TLR9 (forward 5′-GCAAGCTCAACCTGTCCTTC-3′, reverse 5′-TAGAAGCAGGGGTGCTCAGT-3′) and β-actin (forward 5′-AGCCATGTACGTAGCCATCC-3′, reverse 5′CTCTCAGCTGTGGTGGTGAA-3′).

### Western blot analysis

NK cells (1×10^6^) were infected with VV with MOI of 1 for the indicated time periods at 37°C. Western blot analysis was conducted as previously described [35]. Briefly, samples were transferred to a nitrocellulose membrane following separation on SDS-PAGE gels (Bio-rad). Membranes were blotted overnight at 4°C with anti-phospho-p44/42 MAPK (ERK1/2) (Thr202/Tyr204) (E10) Mouse mAb or Phospho-Akt (Thr308) Rabbit pAb (Cell Signaling Technologies), washed three times, probed with an Alexa-Fluor 680-conjugated anti-mouse Ig secondary antibody (for pERK1/2) or an Alexa-Fluor 680-conjugated anti-rabbit Ig secondary antibody (for pAkt) (Molecular Probes), followed by visualizing the Odyssey infrared imaging system (LI-COR). Membranes were then stripped and probed with an anti-total Erk1/2 or anti-total Akt antibody (Cell Signaling Technologies) to serve as a loading control.

### Statistical analysis

A two-sided, two sample student *t*-test with 95% confidence bounds was used for statistical analysis. Data are presented as mean ± sd. All statistical analyses were performed using the SAS/STAT software (SAS Institute, Cary, NC) as we previously described [42].

## Supporting Information

Figure S1 Phenotypic analysis of WT and TLR2^−/−^ NK cells. DX5^+^CD3^−^ NK cells from WT or TLR2^−/−^ mice were stained with anti-CD69, anti-CD62L or anti-KLRG1, as well as their corresponding isotype controls, and subjected to FACS analysis. The percentages of CD69-, CD62L- and KLRG1-postive NK cells among DX5^+^CD3^−^ NK cells are indicated.
(0.14 MB TIF)

Figure S2 Purity of sorted NK cells. Splenic NK cells were first enriched using anti-DX5-PE and PE-microbeads. The DX5^+^ cells were then stained with anti-CD3-FITC and subjected to FACS sorting gated on the DX5^+^CD3^−^ population. The percentages of DX5^+^CD3^−^ vs. DX5^+^CD3^+^ populations before (Pre-sort) and after (Post-sort) sorting are indicated.
(0.11 MB TIF)

Figure S3 The role of TLR2-independent NK cell activation in VV control. (A) Female WT or TLR2^−/−^ mice were depleted of NK cells with anti-NK1.1 antibodies on days −3 and 0 (+anti-NK1.1) or left untreated (Control), followed by infection with VV. 48 h after infection, the ovaries were assayed for viral load. Data represents viral titer ± SD as pfu per ovary. (B) Female WT mice were depleted of NK cells on days −2 with anti-NK1.1 antibodies. On day 0, NK cell-depleted mice were reconstituted with highly purified NK cells, followed by infection with VV. 48 hr after infection, the ovaries were assayed for viral load. Data represents viral titer ± SD as pfu per ovary.
(0.11 MB TIF)

Figure S4 TLR2 expression on NK cells. (A) RNA was isolated from purified DX5^+^CD3^−^ NK cells and subjected to RT-PCR for expression of TLR2, 3, 4, 7, 8, and 9. (B) Purified NK cells were infected with VV (+VV) or left uninfected in the presence of IL2 and IFN-α. 48 h later, cells were stained with anti-TLR2 and subjected FACS. Untreated freshly isolated NK cells (Fresh) were stained with anti-TLR2 or an isotype antibody as controls. The percentages of TLR2-expressing cells are indicated.
(0.33 MB TIF)
