# Supplementary figures and images for: Direct TLR2 Signaling Is Critical for NK Cell Activation and Function in Response to Vaccinia Viral Infection
Source: PLoS Pathog. 2010 Mar 12;6(3):e1000811. doi: 10.1371/journal.ppat.1000811 (PMC2837413; doi:10.1371/journal.ppat.1000811)

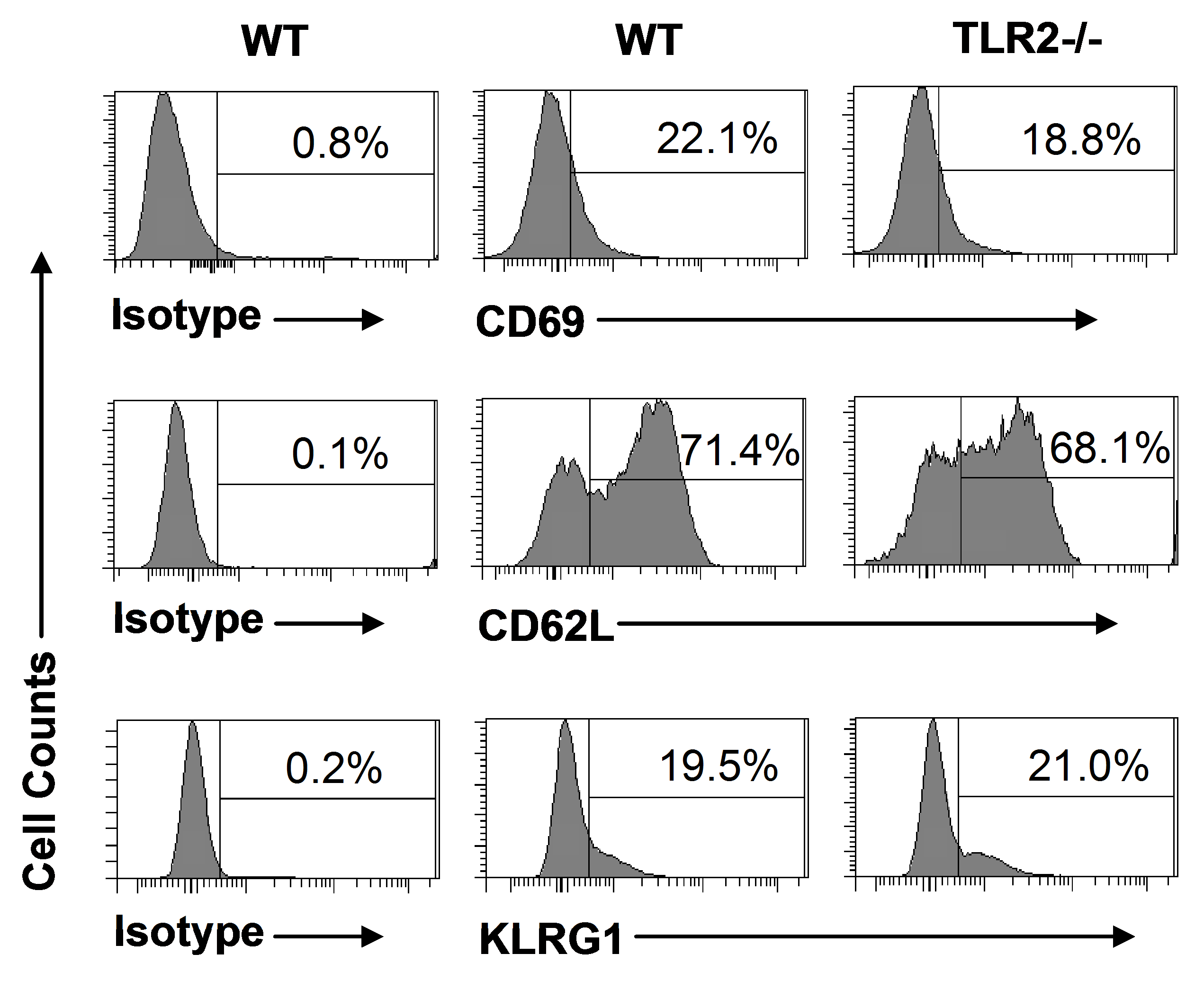

Supplement: Figure S1 — Phenotypic analysis of WT and TLR2−/− NK cells. DX5+CD3− NK cells from WT or TLR2−/− mice were stained with anti-CD69, anti-CD62L or anti-KLRG1, as well as their corresponding isotype controls, and subjected to FACS analysis. The percentages of CD69-, CD62L- and KLRG1-postive NK cells among DX5+CD3− NK cells are indicated. (0.14 MB TIF) [file ppat.1000811.s001.tif]

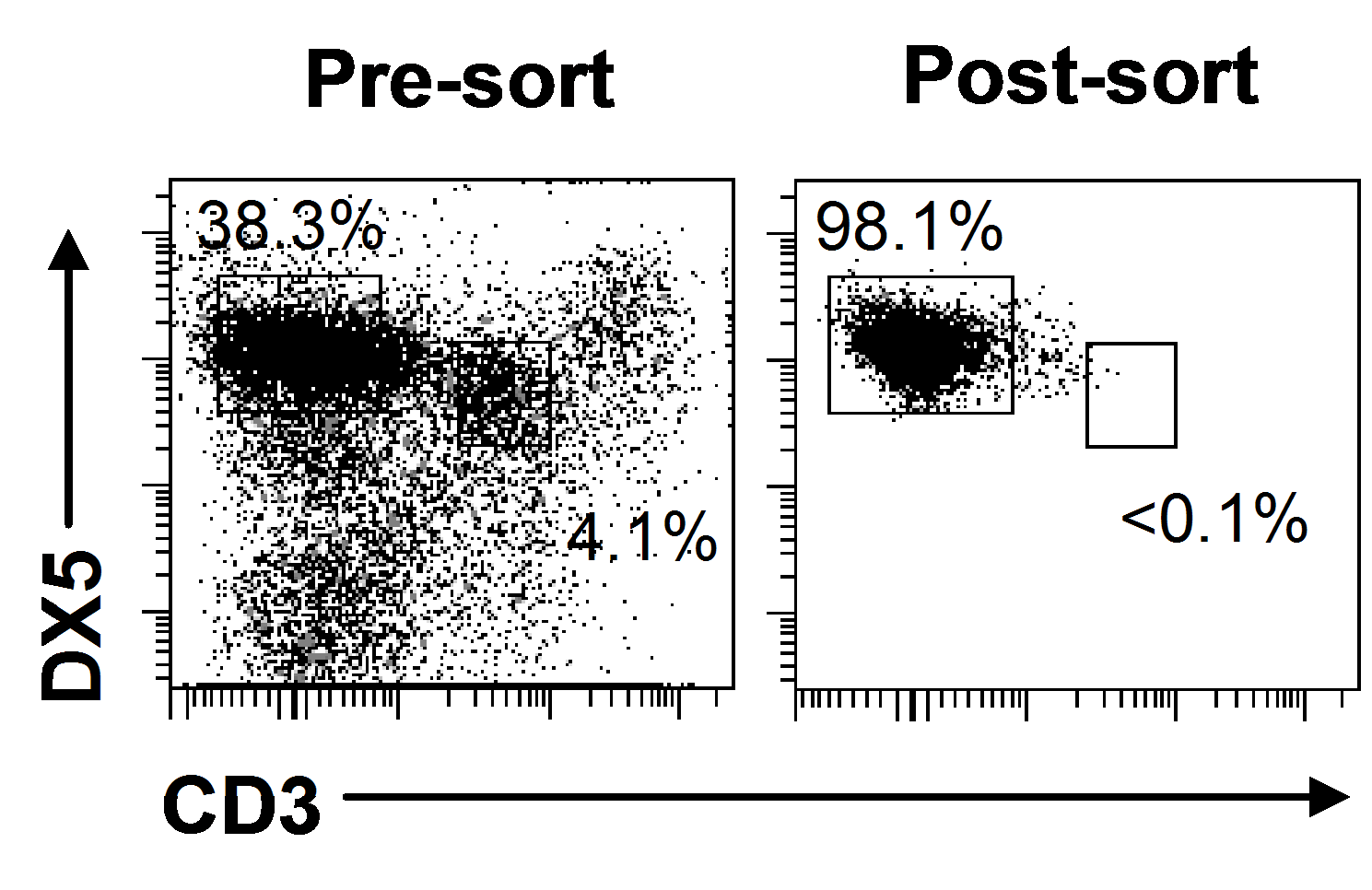

Supplement: Figure S2 — Purity of sorted NK cells. Splenic NK cells were first enriched using anti-DX5-PE and PE-microbeads. The DX5+ cells were then stained with anti-CD3-FITC and subjected to FACS sorting gated on the DX5+CD3− population. The percentages of DX5+CD3− vs. DX5+CD3+ populations before (Pre-sort) and after (Post-sort) sorting are indicated. (0.11 MB TIF) [file ppat.1000811.s002.tif]

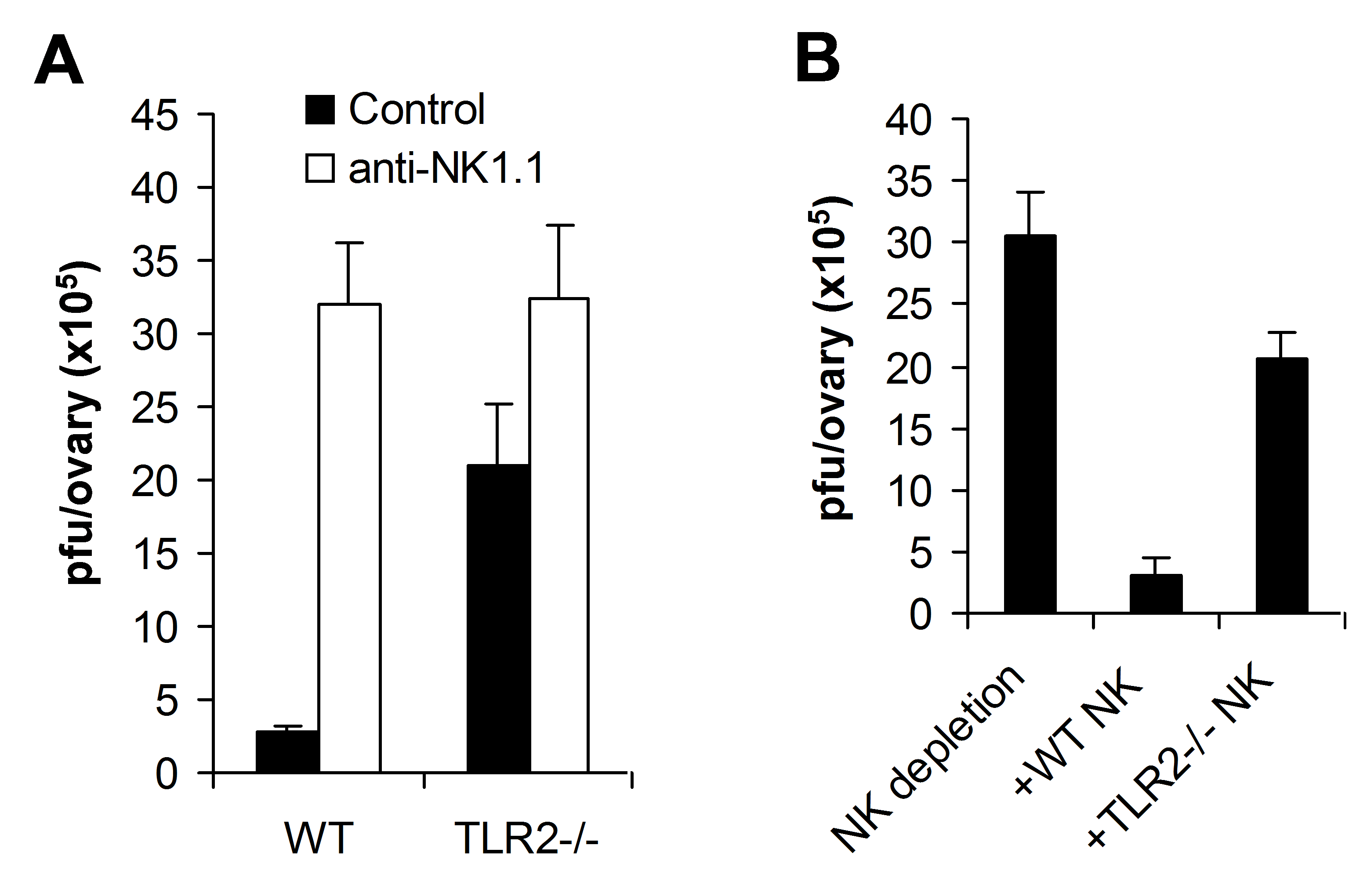

Supplement: Figure S3 — The role of TLR2-independent NK cell activation in VV control. (A) Female WT or TLR2−/− mice were depleted of NK cells with anti-NK1.1 antibodies on days −3 and 0 (+anti-NK1.1) or left untreated (Control), followed by infection with VV. 48 h after infection, the ovaries were assayed for viral load. Data represents viral titer ± SD as pfu per ovary. (B) Female WT mice were depleted of NK cells on days −2 with anti-NK1.1 antibodies. On day 0, NK cell-depleted mice were reconstituted with highly purified NK cells, followed by infection with VV. 48 hr after infection, the ovaries were assayed for viral load. Data represents viral titer ± SD as pfu per ovary. (0.11 MB TIF) [file ppat.1000811.s003.tif]

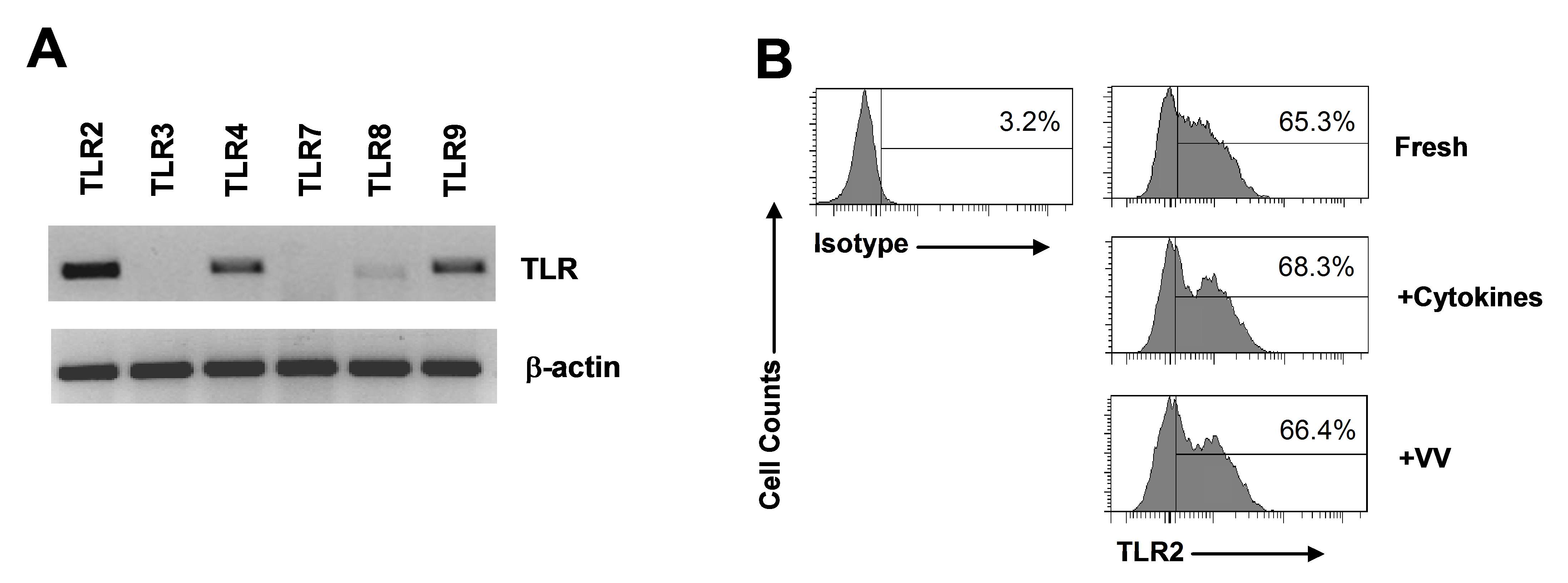

Supplement: Figure S4 — TLR2 expression on NK cells. (A) RNA was isolated from purified DX5+CD3− NK cells and subjected to RT-PCR for expression of TLR2, 3, 4, 7, 8, and 9. (B) Purified NK cells were infected with VV (+VV) or left uninfected in the presence of IL2 and IFN-α. 48 h later, cells were stained with anti-TLR2 and subjected FACS. Untreated freshly isolated NK cells (Fresh) were stained with anti-TLR2 or an isotype antibody as controls. The percentages of TLR2-expressing cells are indicated. (0.33 MB TIF) [file ppat.1000811.s004.tif]
